# Walking with robot-generated haptic forces in a virtual environment: a new approach to analyze lower limb coordination

**DOI:** 10.1186/s12984-021-00823-5

**Published:** 2021-09-09

**Authors:** Gianluca U. Sorrento, Philippe S. Archambault, Joyce Fung

**Affiliations:** 1 School of Physical & Occupational Therapy, McGill University, Montreal, QC Canada; 2 Centre for Interdisciplinary Research in Rehabilitation (CRIR) of Greater Montreal, Jewish Rehabilitation Hospital (CISSS-Laval), Laval, QC Canada

**Keywords:** Gait, Coordination, Locomotor adaptation, Virtual reality, Haptic devices

## Abstract

**Background:**

Walking with a haptic tensile force applied to the hand in a virtual environment (VE) can induce adaptation effects in both chronic stroke and non-stroke individuals. These effects are reflected in spatiotemporal outcomes such as gait speed. However, the concurrent kinematic changes occurring in bilateral lower limb coordination have yet to be explored.

**Methods:**

Chronic stroke participants were stratified based on overground gait speed into lower functioning (LF < 0.8 m/s, N = 7) and higher functioning (HF ≥ 0.8 m/s, N = 7) subgroups. These subgroups and an age-matched control group (N = 14, CG) walked on a self-paced treadmill in a VE with either robot-generated haptic leash forces delivered to the hand and then released or with an instrumented cane. Walking in both leash (10 and 15 N) and cane conditions were compared to pre-force baseline values to evaluate changes in lower limb coordination outcomes.

**Results:**

All groups showed some kinematic changes in thigh, leg and foot segments when gait speed increased during force and post-force leash as well as cane walking. These changes were also reflected in intersegmental coordination and 3D phase diagrams, which illustrated increased intersegmental trajectory areas (p < 0.05) and angular velocity. These increases could also be observed when the paretic leg transitions from stance to swing phases while walking with the haptic leash. The Sobolev norm values accounted for both angular position and angular velocity, providing a single value for potentially quantifying bilateral (i.e. non-paretic vs paretic) coordination during walking. These values tended to increase (p < 0.05) proportionally for both limbs during force and post-force epochs as gait speed tended to increase.

**Conclusions:**

Individuals with chronic stroke who increased their gait speed when walking with tensile haptic forces and immediately after force removal, also displayed moderate concurrent changes in lower limb intersegmental coordination patterns in terms of angular displacement and velocity. Similar results were also seen with cane walking. Although symmetry was less affected, these findings appear favourable to the functional recovery of gait. Both the use of 3D phase diagrams and assigning Sobolev norm values are potentially effective for detecting and quantifying these coordination changes.

## Background

Challenges to balance and mobility commonly persist among individuals with chronic stroke. Hemiparesis of the affected lower limb is often observed during gait leading to a varying degree of biomechanical asymmetry [1] as well as kinematic and spatiotemporal differences between the affected and non-affected limbs [2]. The limited mobility often seen in stroke is linked to a higher risk of falling [3]. Indeed, fall rates over 40% have been reported within the first six months post stroke [4, 5]. Given these high fall rates, improving mobility for independent ambulation continues to pose a challenge to many older individuals with chronic stroke [6].

Various technologies including virtual reality (VR), haptic robotics, and adapted treadmills have all been employed to help address the challenges of regaining walking function post stroke. For example, training in VR settings can lead to improved balance and a reduction in falls [7], while offering an added beneficial effect relative to conventional therapy [8]. The use of VR can also be individualized to the person’s needs for cognitive and motor recovery [9]. In relation to the latter, chronic stroke individuals who trained in VR have shown to change aspects of range of motion (ROM) of lower limb segments [10]. In gait rehabilitation, VR is often used in tandem with treadmill walking. Individuals with chronic stroke have shown they can safely adapt to different walking demands in different virtual environments (VEs) with treadmills [11]. They have also shown changes in paretic limb coordination contributing to a changing posture conducive to forward motion during treadmill walking [12]. In addition, walking to pre-set paces such as on a split-belt treadmill with belts moving at different speeds can also induce gait adaptations with lingering aftereffects [13]. It was also found that individuals with chronic stroke are capable of producing these adaptation effects [14], which can also transfer to overground walking [15]. Other examples of related adaptation and aftereffect mechanisms include gait paradigms featuring perturbation platforms [16, 17].

The addition of haptic inputs can also affect spatiotemporal and biomechanical outcomes [18] as well as balance [19]. For example, the use of haptic plantar stimuli have been used to create gait adaptations on the split-belt treadmill, which can then transfer to overground  walking [20]. Light touch haptic cues, such as the haptic bar coupled with VR, have also been shown to improve gait stability in different walking conditions [21]. Another form of stationary haptic stimuli used with chronic stroke are instrumented handrails, which can improve balance and gait economy [22]. There have also been more dynamic forms of haptic input, such as the instrumented cane [23, 24] and the rehabilitation dog [25], the latter providing some ecological context to the mechanical leash [26] proposed in the current study. When walking overground with a rehabilitation dog, chronic stroke individuals have improved gait speed and reduced gait deviations related to lower limb coordination, even with respect to a cane, over the course of a training protocol [25].

The current study is a direct follow-up from Sorrento et al. 2018, which proposed a novel approach to using VR and treadmill walking with robot-driven haptic tensile forces applied to the hand via a mechanical leash to induce gait adaptation effects. These adaptations can  lead to new strategies for improving important spatiotemporal outcomes in gait rehabilitation. Gait speed was notably increased when either a 10 or 15 N force was applied and was maintained by approximately 0.05–0.1 m/s above baseline immediately after the force was released in chronic stroke and healthy elderly individuals [27]. Similar gait speed increases were also reported in a younger population [26]. Moreover, a similar forward leading haptic tensile force set-up during overground walking has since followed up with similar spatiotemporal effects [28]. Thus, there is growing evidence that walking with haptic tensile forces in the direction of movement increases gait speed.

However, it has been noted that training changes in gait speed in a chronic stroke population can be achieved by reinforcing compensation strategies [29, 30]. Indeed, it has also been suggested that the functional recovery of gait does not necessarily entail restoring a normal gait pattern [31]. For example, gait speed increases seen in robot-assisted gait training may not lead to changes in the symmetry of gait between the paretic non-paretic side [32]. Yet, it is also found that gait speed increases are also met with increases in peak joint angular velocity of lower limb joints [33]. It is therefore necessary to assess the extent to which the gait speed increases previously seen in response to haptic tensile forces also change posture and bilateral coordination. In terms of postural changes, it was suggested that the centre of mass (COM) may displace to the paretic side for some chronic stroke individuals when the haptic force is present and after it is removed [27], possibly further engaging the paretic lower limb. However, little is known to date of the extent to which lower limb coordination of the thigh, leg, and foot segments change in parallel to the spatiotemporal and postural changes as a result of exposure to the haptic leash.

The objective of this study is therefore to  estimate the extent to which changes reported in spatiotemporal gait outcomes due to haptic tensile forces, notably gait speed [26, 27], are also reflected kinematically in the intersegmental coordination of the bilateral lower limbs. It also remains to be seen if potential changes are conducive to both the functional recovery and symmetry of gait in chronic stroke. To address these objectives, a series of kinematic outcomes are presented. Relative changes in limb segment angles of the thigh, leg and foot across leash and cane walking epochs are compared and contrasted in the context of two graphical methods. The first presented is the intersegmental coordination diagram of thigh, leg, and foot segments similar to those previously reported to describe planar covariation under various walking speeds and conditions [34, 35] and specifically for stroke populations [36–38]. From this diagram, changes in bilateral intersegmental surface areas of the combined thigh, leg and foot segments can be compared across epochs and conditions. The other is the 3-dimensional (3D) phase diagram, which offers a visual representation of both angular position and angular velocity of thigh, leg and foot segments. The added velocity component featured in the 3D phase diagram allows for the estimation of Sobolev norm values for each leg, which may provide an evaluation of coordination for both legs. The single metric value calculated for both lower limbs may offer additional insights into the changes in bilateral symmetry as a result of adapting gait to haptic forces. Lastly, mapping the relation between gait speed and intersegmental surface area as well as Sobolev norm values can give a further indication of whether the concurrent changes in coordination resulting from moderate gait speed increases, prompted by haptic tensile forces, are conducive to functional recovery and symmetry.

## Methods

### Participants

Fourteen hemiparetic chronic stroke individuals between the ages of 65 and 80 years old (70.6 y.o. ± 2.9 y.o.) and 14 non-stroke, aged-matched control participants (71.8 y.o. ± 2.7) were recruited for this study (see Table 1). Chronic stroke participants were at least 6-months post stroke at the time of participation and displayed mild to moderate lower limb and foot impairment with Chedoke-McMaster Stroke Assessment scores between 3 and 6. Individuals with cerebellar stroke lesions were excluded from the study. Additional exclusion factors for both chronic stroke and control individuals included any other neurological conditions or musculoskeletal injuries that would impair gait. All participants were required to walk independently at a comfortable pace without pain for at least three minutes. Participants who were unable to habituate to the self-paced treadmill were excluded from the study. As previously reported [27], chronic stroke individuals were further stratified into lower function (LF) and higher function (HF) subgroups based on a 0.8 m/s overground gait speed threshold. Stratification was deemed necessary as lower function individuals may exhibit changes (i.e. gait speed) relative to smaller baseline values [39] and tend to walk with assistive devices (see Table 1) compared to higher function individuals. The gait speed cut-off was determined by the 0.8 m/s overground gait speed for full community dwelling [40]. Before participation in the study, all participants were presented with a consent form approved by the ethics committee of the Centre for Interdisciplinary Research in Rehabilitation of Greater Montreal (CRIR). All participants reviewed the form and provided written informed consent to participate.

**Table 1:** Participant demographics

Factor	Lower function chronic stroke (LF)	Higher function chronic stroke (HF)	Control group (CG)
Sample size (n)	7	7	14
Gender (M/F)	5/2	5/2	7/7
Age (years)	70.4 ± 3.2	70.9 ± 2.9	71.8 ± 2.7
Leash/cane hand (left/right)	6/1	2/5	5/9
Gait speed—overground (m/s)	0.55 ± 0.18	1.04 ± 0.26	1.33 ± 0.23
Chronicity (years)	> 0.5	> 0.5	N/A
Use of assistive device—overground	6	1	0

Demographics table for lower function chronic stroke (LF), higher function chronic stroke (HF) and healthy control group (CG) individuals. The LF and HF subgroups were determined based on a 0.8 m/s overground walking speed threshold. Assistive devices for daily overground walking included the use of canes and walkers. Individuals in the LF and HF groups held the leash or cane in the non-paretic hand, while individuals in the CG group held the leash or cane with the preferred dominant hand.

**Fig. 1:**
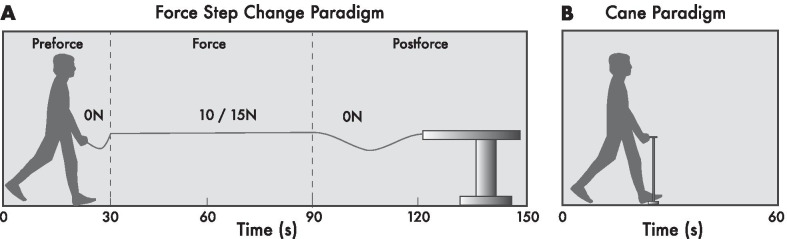
Haptic leash and instrumented cane paradigms. **a** In the force step change paradigm (Sorrento et al. [26]), participants initially walked with 0 N of leash tension in the pre-force epoch. After ~ 30 s a haptic tensile force of either 10 or 15 N was delivered to the hand in the force epoch. The haptic force was then released after  60 s as participants continued to walk in the post-force epoch for another 60 s. **b** In the cane paradigm, participants walked on the self-paced treadmill with a stationary instrumented cane for a minimum of 60 s

### Procedure and apparatus

Average 10-m overground walking speed was measured for all participants. These values were used to stratify the lower (< 0.8 m/s) and higher (≥ 0.8 m/s) function chronic stroke participants and to establish a target walking speed on the self-paced treadmill during the walking habituation phase prior to the experiment. The participants’ anthropometrical data were recorded and they were subsequently fitted into a full body harness mounted to the ceiling. A full set of reflective markers were fixed with skin tape to body landmarks, follwing the the Vicon ‘Plug-In Gait’ model. An additional six markers were placed on the treadmill frame and haptic leash. Data capture consisted of six Vicon cameras recording at a 120 Hz frame rate. The system was recalibrated prior to every recording.

A proportional-integral-derivative (PID) controller was used to drive a 0.8 m wide × 1.7 m long self-paced treadmill. The PID controller can modulate treadmill speed changes based on the participant's real-time distance. This was possible with the use of a potentiometer registering distance changes from an extensible cord tethered to the participant’s harness. The cord displacement during locomotion, relative to its calibrated starting position, is continually monitored by a micro-controller that converts distance and velocity signals that update the VE powered by the CAREN-3 platform [11]. The VE scene was synchronized with the self-selected speed of the participant. To provide an ecological context, the scene featured a city setting and a dog avatar to enhance the experience of walking with a leash [26]. The scene was rear-projected from approximately 2.5 m away onto a 2.5 m × 3 m projection screen, while the participant walked approximately 1.5 m in front of the screen on a pre-marked calibrated starting position on the treadmill. A haptic robotic arm (HapticMaster) was used to deliver haptic tensile forces to a hand-held leash [26]. Custom developed software was used to control the step-wise changes of forward tensile haptic forces of 0–10 or 15 N. A custom-made end effector was attached to the robotic arm and connected a steel, stretch-resistant cord leash to the handle, via a pulley system [26].

### Force step change and instrumented cane paradigms

Before the experiment, participants were habituated to walking on the self-paced treadmill with their natural gait pattern. They were instructed to walk at a comfortable pace for all trials. After the free walk habituation phase, participants began the force step change paradigm by walking in the VE for 30 s in the pre-force epoch holding the mechanical leash with the non-affected hand (LF and HF subgroups) or the dominant hand (CG group, see Fig. 1). A 3 N residual force was fed into the leash system from the robot. This force was calibrated a priori to keep the leash primed for the force step change and avoid a delayed mechanical pull on a slack leash at force onset, while still avoiding any perceived force in the hand. No other assistive device was allowed for leash trials. After 30 s, the robotic arm was activated sending an instantaneous force through the pulley system to produce either 10 or 15 N of tensile force to the leash handle as the participant continued to walk in the force epoch for another 60 s. Finally, the leash tension was released from the hand and the participant continued to walk a final 60 s in the post-force epoch. The duration of each force step change paradigm was approximately 150 s. One trial of 10 and 15 N conditions was required and only steady-state walking portions of the paradigm were used for analysis. For the separate instrumented cane paradigm, participants walked holding the fixed instrumented cane [23] in the non-affected (LF and HF) or dominant (CG) hand  once for a minimum of 60 s (see Fig. 1).

### 3D intersegmental coordination diagram

**Fig. 2:**
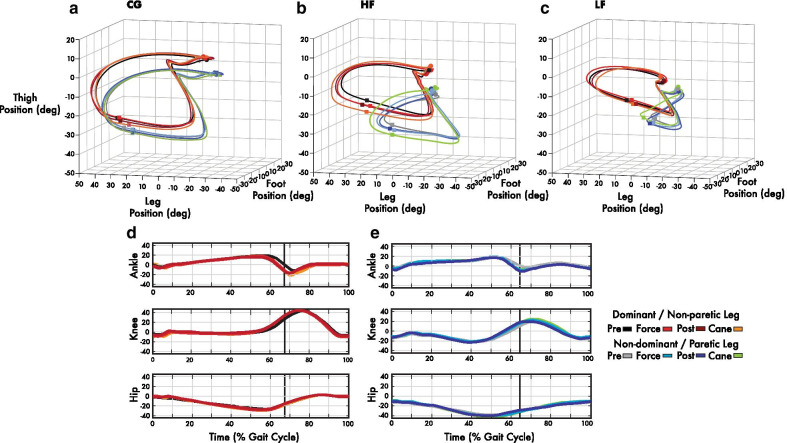
Bilateral 3D intersegmental coordination diagrams. The diagrams illustrate intersegmental surface area for the dominant/non-paretic and non-dominant/paretic thigh, leg, and foot segments in 3D space across an average gait cycle for a representative **a** control (CG), **b** higher function (HF) and **c** lower function (LF) chronic stroke participant in pre-force, force, and post-force leash epochs and the cane condition. The dots and squares on the top and bottom of trajectories indicate the points of heel strike and toe lift, respectively. **d**, **e** Average hip, knee, and ankle joint elevation angles of the representative HF individual for both **d** non-paretic and **e** paretic legs during leash epochs and cane conditions. Knee and ankle angle increases can be seen in the stance to swing transition (vertical line) for force, post-force, and cane epochs as well as slight increases in general ROM. Positive values indicate flexion/dorsiflexion and negative values indicate extension/plantarflexion, relative to neutral position

The 3D intersegmental coordination diagram provides a detailed illustration of the thigh, leg, and foot segment positions throughout an average gait cycle in a common angular space. These segments were taken from reference angles at the hip (relative to vertical), knee and ankle angles (see Fig. 2). The orientation of the plane was obtained from the minimum summed perpendicular distance from the intersegmental angular position trajectory (see Fig. 2a–c). Superimposing left and right lower limb trajectories also provides some indication of the kinematic symmetry of both legs, since the orientation of the planes correspond to the relative contributions of the three limb segments. This approach can allow for visually detecting whether there are relative changes in bilateral coordination across epochs in the leash and cane conditions.

**Fig. 3:**
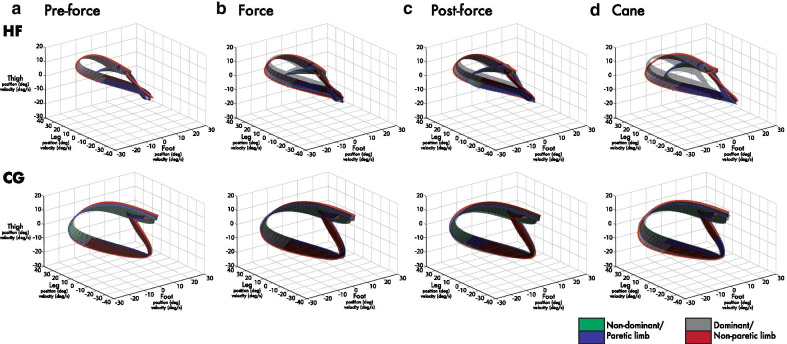
Bilateral 3D phase diagrams. Representative 3D phase diagrams of average gait cycles for a representative HF and CG participant during the **a** pre-force, **b** force, **c** post-force, and **d** cane conditions. **b–d** The darker outline of the pre-force 3D phase diagram (from **a**) is superimposed for direct comparison with force, post-force and cane diagrams. The intersegmental angular trajectories (outer traces) are joined by perpendicular angular velocity ribbon traces. Blue to green (left leg) and red to grey (right leg) colour changes correspond to stance and swing phases, respectively. The circles and squares correspond to the moment of heel strike and toe lift, respectively. Some increases in angular velocity traces (thickening of ribbon traces) can be seen in the gait cycle such as the toe lift and during the swing phase in force, post-force, and cane 3D phase diagrams (**b**–**d**) for the HF individual in particular. Greater overlap with the pre-force trace is seen with the CG individual in comparison (**b**–**d**)

### 3D phase diagrams

A more recent approach to illustrate lower limb coordination is the 3D phase diagram. It builds on the previous coordination plot, with the addition of a continuous angular velocity profile throughout the entire gait cycle, accounting for the thigh, leg and foot segments. Viewing the entire angular velocity profile can be an informative tool when investigating specific gait cycle events undergoing angular velocity changes as a result of concurrent changes in gait speed and other spatiotemporal variables. The magnitude of the angular velocity is plotted perpendicular to the angular position trajectory, thus making an angular velocity trace visible in the 3D phase diagram. This is achieved by multiplying the angular velocity by a transformation matrix seen in Goussev [41] given below to display the corresponding perpendicular angular velocity magnitude, visible throughout the gait cycle [41].

1 $$A = \left( {\begin{array}{*{20}c} 0 & { - 1} & { - 1} \\ 1 & 0 & { - 1} \\ 1 & 1 & 0 \\ \end{array} } \right)$$

### Sobolev norm values

Coordination and symmetry changes of the lower limb can be potentially achieved by quantifying a single value for both the angular position and the corresponding angular velocity throughout the gait cycle. These two components are conveniently made available by the 3D phase diagram. The approach that allows for such a measure is the mean Sobolev norm value. Obtaining a value for both left and right lower limbs for each walking epoch and condition is rather novel in gait and is a potentially beneficial method of obtaining a metric for evaluating bilateral lower limb symmetry. The mean Sobolev norm (S) is computed as the square root of the mean squared sum of the angular position 3D trajectory trace (γ) and its corresponding angular velocity ($\dot{\gamma }$) both taken per frame throughout the gait cycle, as previously described [42].

2 $$S = \sqrt {{{\left( {\sum\limits_{i = 1}^{n} {\gamma_{i}^{2} } + \sum\limits_{i = 1}^{n} {\dot{\gamma }_{i}^{2} } } \right)} \mathord{\left/ {\vphantom {{\left( {\sum\limits_{i = 1}^{n} {\gamma_{i}^{2} } + \sum\limits_{i = 1}^{n} {\dot{\gamma }_{i}^{2} } } \right)} {\left( {n - 1} \right)}}} \right. \kern-\nulldelimiterspace} {\left( {n - 1} \right)}}}$$

### Data analysis

Custom software was developed to control and to record haptic tensile forces from the HapticMaster robotic arm. To synchronize Vicon and HapticMaster systems, an analog pulse was simultaneously sent from the CAREN system to both systems. This synchronization was necessary for the analysis of data across motion capture and robot platforms and the demarcation of critical time points for force step change onset and offset, which defined paradigm epochs.

Motion capture data from Vicon was analyzed using custom Matlab programs. The generalized estimated equations (GEE) analysis was the statistical model used for gait speed, intersegmental joint angles, intersegmental area, and Sobolev norm outcomes. A main interaction accounted for conditions (10 N, 15 N, and cane), epochs (pre-force, force, post-force) and side (dominant/non-paretic and non-dominant/paretic) for LF, HF, and CG groups. Tests for data normality for all outcomes were conducted and significance was accepted at p < 0.05. Post-hoc comparisons were also set at p < 0.05. Comparisons were also conducted between groups and for the bilateral lower limbs. Finally, a bivariate Pearson’s correlation was used to investigate both intersegmental surface areas and Sobolev norm values with respect to gait speed for both dominant/non-paretic and non-dominant/paretic sides.

## Results

### Gait speed

Both LF and HF individuals on average increased gait speed by over 0.04 m/s and 0.11 m/s, respectively during the force epoch relative to the pre-force baseline speed (see Table 2) [27]. Gait speed was then maintained in both LF and HF groups by as much as 0.07 m/s after force removal during the post-force epoch (see Table 2). These changes were comparable to gait speed increases in the cane condition (0.04–0.05 m/s), relative to pre-force levels. The CG participants also increased gait speed by as much as 0.13 m/s and 0.14 m/s for the 10 N force and post-force epochs compared to pre-force baseline values. Walking with the cane, compared to without, produced an average 0.04 m/s gait speed change. Statistical analysis revealed a statistically significant interaction for gait speed between epochs (pre-force, force and post-force) and conditions (10 N, 15 N, and cane) for CG, HF, and LF groups (p < 0.05). Pairwise comparisons determined significant changes for the CG group during 10 and 15 N force epochs, the HF group during the 10 N force epoch and the LF group during the 10 and 15 N post-force epoch, all relative to their respective pre-force gait speed baselines (p < 0.05, see Table 2). Also detected were significant differences between CG and LF groups across 10 and 15 N force and post-force epochs and cane condition (p < 0.01).

**Table 2:** Group averages for gait speed, intersegmental trajectory area, and Sobolev norm values

Group	Outcome	Preforce	10 N force	Postforce	Preforce	15 N force	Postforce	Cane
	Gait speed (m/s)	0.27 ± 0.12	0.31 ± 0.13	0.31 ± 0.14*	0.24 ± 0.08	0.29 ± 0.09	0.30 ± 0.10*	0.31 ± 0.15
LF	Area (deg^2^)	6.2 ± 3.0/1.6 ± 1.1	7.6 ± 4.0/1.9 ± 0.9	7.7 ± 4.2/1.9 ± 0.9	5.9 ± 2.4/1.6 ± 0.7	6.9 ± 2.6/1.9 ± 1.1	7.3 ± 2.8/2.2 ± 1.0	6.4 ± 3.5/1.9 ± 0.8
	Sobolev norm	26.6 ± 6.9/14.4 ± 4.5	28.6 ± 7.7/15.8 ± 3.1	28.6 ± 9.5/16.1 ± 3.0	26.2 ± 6.9/13.4 ± 3.2	27.6 ± 6.1/15.3 ± 3.8*	28.3 ± 7.3/17.0 ± 4.3*	29.1 ± 9.5*/15.9 ± 3.5*
	Gait speed (m/s)	0.63 ± 0.32	0.74 ± 0.35*	0.68 ± 0.36	0.66 ± 0.36	0.77 ± 0.38	0.73 ± 0.39	0.68 ± 0.36
HF	Area (deg^2^)	12.8 ± 7.5/8.9 ± 6.9	14.2 ± 7.9/9.8 ± 7.3	13.3 ± 8.2/9.5 ± 7.0	13.1 ± 7.9/9.0 ± 7.4	15.0 ± 8.4*/9.5 ± 7.5	15.5 ± 8.8/10.2 ± 7.7	13.4 ± 8.7/9.7 ± 7.4
	Sobolev norm	38.0 ± 12.9/31.5 ± 14.4	41.5 ± 14.2/33.7 ± 14.7	40.6 ± 18.2/33.1 ± 14.6	37.9 ± 15.2/31.3 ± 15.6	41.7 ± 15.6*/33.6 ± 15.3	41.1 ± 15.6/34.2 ± 14.7	38.5 ± 15.5/32.4 ± 15.3
	Gait speed (m/s)	1.00 ± 0.34	1.13 ± 0.30*	1.14 ± 0.30	1.07 ± 0.33	1.19 ± 0.34*	1.15 ± 0.35	1.08 ± 0.37
CG	Area (deg^2^)	19.4 ± 5.9/19.3 ± 7.0	21.1 ± 5.8/21.6 ± 7.1*	22.6 ± 6.9/22.0 ± 8.0	20.4 ± 5.4/20.5 ± 7.2	21.0 ± 6.4*/22.6 ± 7.6	21.9 ± 5.3/22.6 ± 8.9	20.9 ± 7.3/21.7 ± 7.7
	Sobolev norm	47.5 ± 8.4/47.3 ± 9.7	51.3 ± 7.2*/50.9 ± 8.6*	51.6 ± 7.0/50.7 ± 8.0*	48.5 ± 6.8/48.9 ± 8.8	50.8 ± 6.3*/51.6 ± 8.1*	51.8 ± 7.5/51.3 ± 8.6	49.8 ± 8.9/49.1 ± 9.1

**a**: Average and standard deviation values for gait speed, lower limb intersegmental surface area, and Sobolev norm values for nonparetic/paretic (LF and HF) and dominant/non-dominant (CG) sides for pre-force, force, post-force epochs in 10 and 15 N leash conditions as well as the cane condition. Statistical significance is denoted by * (p < 0.05) and is relative to pre-force values

### Average bilataral lower limb joint angles

Changes in average angles across the entire gait cycle relative to the pre-force levels were generally small and mixed across epochs. However, there was some evidence of average increases in ankle and hip angles. For example, the HF group slightly increased ankle and hip angles by an average of 0.4° and 0.8° for the paretic side during the 10 N condition. These increased angle changes were maintained in the post-force epoch (ankle: 0.7°: hip: 1.1°). Mixed results with mostly smaller magnitudes were also observed for the LF and CG groups as were the cane conditions across groups. Statistical analysis revealed some significant interactions for the bilateral ankle, knee, and hip angles across all conditions (p < 0.01). Pairwise comparisons identified bilateral statistical differences for the ankle joint for both HF (non-dominant) and CG (bilateral) groups in the 15 N condition between pre-force and force epochs (p < 0.05) and for the LF group during the 10 N post-force relative to the pre-force epoch (non-dominant, p < 0.05). Average knee joint angles revealed significant changes for the LF (10 and 15 N) and HF (15 N) force and post-force epoch, while the CG group also revealed significant bilateral force (10 N) and non-dominant 10 and 15 N post-force and cane comparisons. The hip joint also showed significant changes for the LF (10 N force and bilateral 15 N post-force) and HF (15 N paretic) force epoch, while the CG group showed significant dominant side post-force (10 N) and non-dominant 15 N force and cane comparisons, relative to the pre-force epoch. A closer examination within the gait cycle of the HF individual featured in Fig. 2d, e (15 N force) reveals an increase in paretic and non-paretic ankle plantarflexion by as much as 6.8° and 10.7°, respectively, as well as 5° and 7.7° of knee flexion during force and post-force epochs, relative to pre-force epoch, at the transition from the stance to swing phase (see Fig. 2d, e). Overall, the HF individual exhibited increases in ROM across the gait cycle for the paretic hip, knee, and ankle with changes of 4.2°, 3.3°, and 6.9°, respectively (Fig. 2e).

### Bilateral intersegmental lower limb surface area

**Fig. 4:**
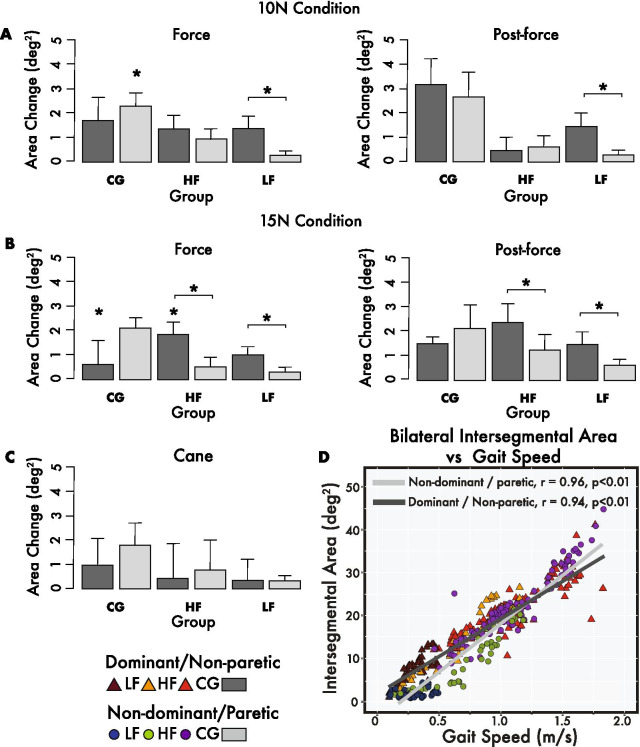
Intersegmental trajectory area changes. **a**–**c** Average changes for bilateral intersegmental surface areas for all LF, HF, and CG participants during **a** 10 N and **b** 15 N force and post-force epochs as well as the **c** cane condition relative to respective pre-force epochs. **d** Intersegmental surface area vs gait speed correlations for dominant/non-paretic and non-dominant/paretic legs. Provided are r values and fitted lines representing all data, while data points are subdivided for LF, HF, and CG groups during pre-force, leash (force, post force), and cane data, respectively. Statistically significant original values are denoted by * (p < 0.05). Significant changes are relative to pre-force levels as well as bilateral comparisons (above lines)

The average intersegmental trajectory surface areas for LF, HF, and CG groups (see Table 2, Figs. 2a–c and 4a–c) on average showed modest yet consistent increases during force and post-force epochs and cane condition relative to the pre-force epoch. For example, the average changes in intersegmental area for the LF subgroup during the 15 N force and post-force epochs for the paretic limb were 0.3 deg^2^ and 0.6 deg^2^, respectively, while the cane condition also yielded a 0.3 deg^2^ change. The HF group on average increased this area by 0.5 deg^2^ and 1.2 deg^2^ for the 15 N force and post force epochs and 0.8 deg^2^ during the cane condition. This would suggest that on average the paretic leg reached a slightly larger range of motion (ROM) when the force was present and remained this way after the force was removed. The CG group also increased intersegmental area of the non-dominant (i.e. non-leash side) leg by 2.1 deg^2^ for the 15 N force and post-force epochs and by 1.8 deg^2^ for the cane condition (see Figs. 2a and 4a–c). A statistically significant main interaction (p < 0.01) was seen with pairwise differences (p < 0.05) in intersegmental area for both HF (non-paretic) and CG (dominant) groups in the 15 N force epoch, as well as 10 N force for CG (non-dominant) individuals relative to pre-force values (See Table 2). Significant bilateral differences between the CG and LF groups for both force and post-force values were also found for the 10, 15 N, and cane conditions (p < 0.05). Further bilateral comparisons revealed statistically significant differences between non-paretic and paretic sides for the LF 10 N force and post-force epochs and both the LF and HF for the 15 N force and post-force epochs (see Fig. 4a, b). Additionally, a bivariate analysis (Pearson’s) revealed a strong positive correlation between the intersegmental surface area and gait speed across all groups with the non-dominant (CG) or paretic leg (LF and HF), r (194) = 0.96, p < 0.01, and the dominant or non-paretic leg (r (194) = 0.95, p < 0.05).

### Average bilateral Sobolev norm values

**Fig. 5:**
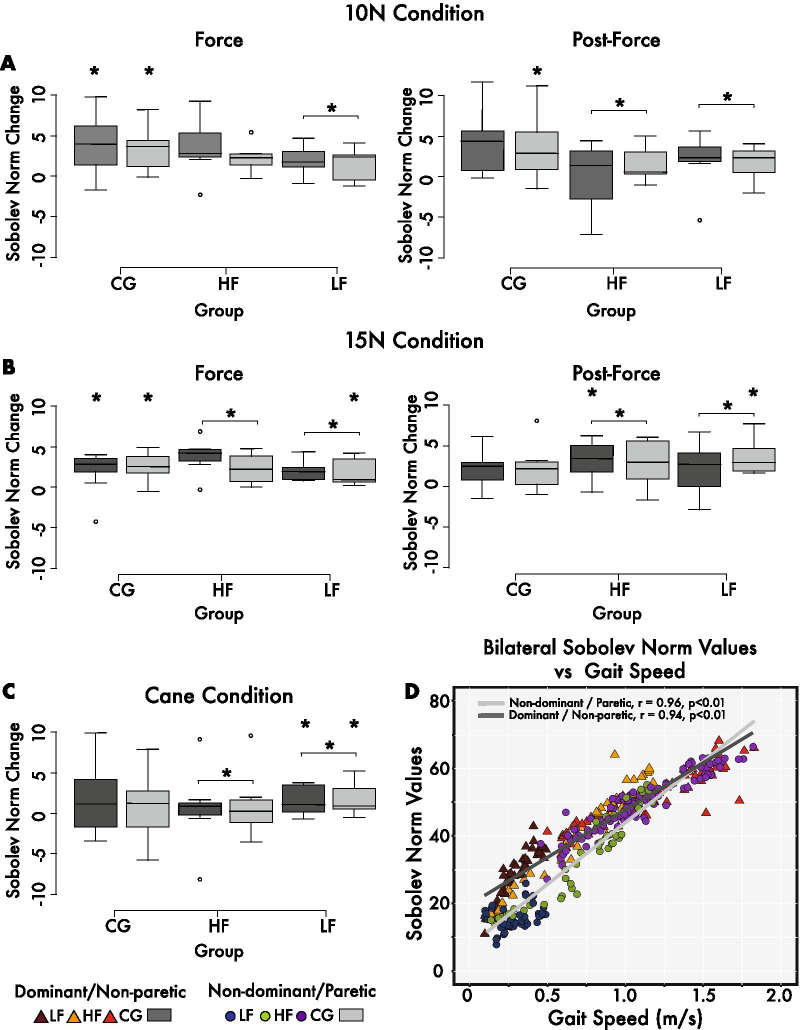
Sobolev norm value changes. **a–c** Box and whisker plots illustrating median and interquartile ranges for Sobolev norm value changes of the dominant/non-paretic and non-dominant/paretic legs for all LF, HF, and CG participants. Changes for force and post-force epochs as well as the cane condition are relative to respective pre-force epoch norms. **d** Sobolev norm values vs gait speed correlation plots for dominant/nonparetic and nondominant/paretic legs. Provided are r values for the bilateral lower limbs across all data and subdivided for LF, HF, and CG groups during pre-force, leash (force and post-force), and cane data. Statistically significant original values relative to pre-force as well as statistically significant bilateral comparisons are denoted by * (p < 0.05)

The Sobolev norm values were compared between epochs and groups for both sides. The increases in both the intersegmental surface area (Fig. 2a–c) and the modest increases in angular velocity during the transition from stance to swing phase and aspects of the swing phase (Fig. 3b–d) contributed to the changes in Sobolev norm values, which on average tended to increase bilaterally for all three groups in the leash condition (see Table 2 and Fig. 5a–c). The average change for the HF subgroup paretic side during the 10 N force epoch was 2.2 and later maintained a 1.6 change during the post-force epoch. Similar changes were seen during the 15 N condition, as the Sobolev norm increased by 2.3 during the force epoch and 2.9 in the post-epoch epoch. The paretic leg value tended to decrease during the cane condition by 1.0. The CG group showed similar changes of 3.6 and 3.4 in the 10 N force and post-force epochs, respectively, for the non-dominant leg. The cane condition also resulted in a slight increase of 0.4 on the non-dominant side (see Fig. 5c). The relative difference in Sobolev norm values between the non-paretic and paretic side remained relatively unchanged except for slight decreases of 0.6 and 1.5 for the LF subgroup during the 15 N force and post-force epochs relative to the pre-force epoch. Statistical analysis revealed a significant main interaction (p < 0.01). Specifically, the LF group showed statistically significant changes for the paretic leg in the 15 N force and post-force epochs and bilaterally for the cane condition relative to pre-force levels. The HF group showed non-paretic changes during the 15 N force condition (p < 0.05, see Table 2 and Fig. 5a–c). The CG group also revealed significant changes in both 10 and 15 N force and the 10 N post-force epoch compared to the pre-force epoch (see Fig. 5a–c). There were also significant bilateral differences between the CG and LF groups for force and post-force during the 10 and 15 N conditions and the dominant/non-paretic cane condition (p < 0.05). Statistically significant bilateral limb comparisons were seen for both HF and LF groups for 10 and 15 N force and post-force epochs as well as the cane condition (p < 0.05, see Fig. 5a–c). A strong positive correlation was also seen between bilateral Sobolev norm values and gait speed across groups with r (194) = 0.96, p < 0.01 for the non-dominant/paretic leg and r (194) = 0.94, p < 0.01 for the dominant/non-paretic leg (see Fig. 5d).

## Discussion

The purpose of this study was to evaluate the extent of kinematic changes in bilateral lower limb coordination in response to continuous haptic tensile forces applied to the hand. The findings were presented in light of evidence previously reported for adaptation and post-adaptation effects seen in gait speed and other spatiotemporal outcomes during the same paradigm in chronic stroke and non-stroke young and elderly populations [26, 27]. In the process, 3D phase diagrams and Sobolev norm values were presented as potential analytical techniques. Both provide an added perspective to kinematic changes seen in angular position and velocity of the lower limb, while spatiotemporal changes were previously observed in the same paradigm [26, 27].

As previously seen, all three groups increased gait speed during the force and post-force epoch with CG and HF groups walking as fast or faster with the leash compared to the cane [27]. These results corroborate with previous findings where a rehabilitation dog was compared to the cane overground [25] and overground walking with haptic cues [28, 43]. Both CG and HF groups increased gait speed by 0.11–0.13 m/s, which may be considered on the lower end of clinically meaningful change [44]. They also maintained on average of 60% of the initial force epoch change during the post-force epoch. The LF group showed the least amount of gait speed increase during leash and cane conditions with respect to pre-force levels, and is perhaps expected that the LF group would yield smaller changes given the lower baseline speed [39]. Nonetheless, on average the LF group was able to carry-over the average changes from the force epoch for the entire 60 s duration of the post-force epoch.

The increases in gait speed invited further examination of coexisting changes in lower limb intersegmental coordination. Average angle changes of the hip, knee and leg segments across the gait cycle were modest and varied across groups and epochs. The HF group did show some evidence of increasing the paretic ankle angles throughout the gait cycle by as much as 0.7° in the 10 N condition. This would suggest that the paretic foot took on a slightly greater dorsiflexion throughout the gait cycle when adapting gait to the haptic force and the subsequent aftereffect. This was was exhibited by the HF individual illustrated in Fig. 2e. However, giving the mixed results one should consider whether the increases in gait speed may have been facilitated by other mechanisms to compensate for the coordination patterns at the hip, leg, and ankle joints [29, 30]. Moreover, the HF individual in Fig. 2e revealed greater plantarflexion during the transition from stance to swing phases with exposure to the leash during force and post-force epochs comparable to cane walking. This should be considered in light of findings suggesting plantarflexion weakness in chronic stroke can impact gait speed [45]. At the same time, the HF individual in Fig. 2e displayed similar changes for peak knee flexion of the paretic side during force and post-force epochs, which was also comparable to the cane condition and may be considered just short of a minimal detectable change levels as reported in healthy individuals [46]. However, it remains to be determined if these changes can persist and increase over the course of training. A closer look at intersegmental limb coordination outcomes can provide more insight into how these changes affect both intralimb coordination and bilateral symmetry.

Elevation angles of the thigh, leg and foot limb segments form a characteristic ellipse trajectory on a plane in 3D space [34]. However, It has been noted that not all types of locomotion necessarily exhibit planar covariation [35]. The bilateral trajectories of intersegmental planar covariation were mostly preserved in relation to the orientation plane (see Fig. 3) for both stroke and control participants when the haptic leash or cane was used. However, relative to the CG trajectories, the LF and  HF individuals revealed distortions in trajectory loops when comparing the paretic leg to the non-paretic leg (see Fig. 2a–c), similar to those described by other authors [36, 38]. Yet, it was found in this study that intersegmental surface areas did marginally increase implying a slightly greater ROM during the force and post-force epochs relative to the pre-force epoch for all groups and for both non-paretic and paretic legs and is illustrated with the HF individual (See Fig. 2a–e). In addition, these changes tended to remain as an aftereffect. Further investigation revealed a strong positive correlation between gait speed and bilateral intersegmental areas across the entire data set (r = 0.96, < 0.01, see Fig. 4d). The LF group had a considerably less positive correlation (r = 0.28) for the paretic side suggesting that lower gait speed increases are met with moderate changes in intersegmental surface area of the paretic leg. However, on closer inspection there was almost no correlation between gait speed and the paretic area during the pre-force (r = 0.07), but did increase for force and post-force epochs (r = 0.20) and the cane (r = 0.77). This does not seem to be the case for the HF group where paretic leg correlations remained mostly above r = 0.9 for leash and cane conditions. A similar result was seen in the CG group. This difference would suggest that changes in intersegmental surface area are sensitive to gait speed. This would also suggest further that the planar covariation is seen at different gait speeds [34, 47] and with haptic tensile force and cane walking conditions, just as its been reported and in other walking conditions such as obstacle clearance [37].

Intersegmental surface area increases in tandem with gait speed increases invites a closer look into the pace in which lower limb trajectory progresses throughout the gait cycle. The 3D phase diagram presented an informative means of additionally expressing angular velocity during stance and swing phases of the gait cycle. The 3D phase diagram can be an informative tool to illustrate angular velocity changes in the gait cycle, particularly in light of modest changes to global joint angles and intersegmental surface areas. The increases found in intersegmental surface area (See Fig. 2a–c and Table 2) were also met with slight increases of angular velocity throughout the gait cycle (see Fig. 3b–d). The small increases in thickness of the ribbons in Fig. 3b–d for force, post-force and cane epochs with respect to the pre-force epoch, particularly in the shift from stance to swing and portions of the swing phase (see blue and red squares in Fig. 3b–d) highlight the small angular velocity increase. For example, in Fig. 3b–d increases in angular velocity were detected at toe lift (square) by the slightly thickened angular velocity ribbon for the paretic leg during post-force epochs particularly for the HF group. This would suggest an increased push-off effect driving the paretic leg into the swing phase due to walking with the haptic force and would be consistent with the plantarflexion and knee flexion increases seen at the same point of the gait cycle (See Fig. 2d, e). A similar thickening of the angular velocity band was also seen during various points of the swing phase (Fig. 3b, c). However, further kinetic analysis would help substantiate this effect.

Finally, the Sobolev norm factoring both the angular limb position and angular limb velocity in a theoretical Sobolev space produced a metric composed of spatial and temporal components that may help address the extent to which both legs are kinematically symmetrical. Both LF and HF subgroups tended to increase Sobolev norm values by as much as 2.3 and 2.9 during both force and post-force epochs for both paretic and non-paretic limbs (see Table 2 and Fig. 5a, b). The LF group, on average reduced the bilateral norm differences between limbs by 0.6 and 1.5 for the 15 N force and post-force epochs (see Table 2). This last finding, while limited in evidence, would suggest that the haptic leash can potentially promote greater symmetry between the paretic and non-paretic limbs. However, given the one-day training provided in this study, the bilateral norm values measured would suggest that the asymmetry between the non-paretic and paretic side was not significantly reduced. A high correlation between the Sobolev norm values and gait speed across groups was also an informative finding. It would suggest that both the modest angular position and velocity increases depicted in intersegmental area and 3D phase diagrams contributed to greater gait speeds found in elderly chronic stroke and control groups (see Table 2). These findings are further substantiated by previous reports where under the same leash conditions younger individuals increased gait speed as evidenced by greater stride lengths and reduced double limb support times [26].

Both dominant/non-paretic and non-dominant/paretic sides displayed strong correlations across groups and epochs (r = 0.94, P < 0.01, and r = 0.96, p < 0.01, respectively). Similar to the intersegmental surface area and gait speed correlation, Sobolev norm values of the LF paretic side showed a positive, yet considerably lower correlation (r = 0.35) with gait speed when compared to CG (r = 0.94) and HF (r = 0.96) groups. This perhaps may be indicative of the different physical capacity of the LF group with respect to CG and even HF groups. Specifically, the lower norm values seen in the LF group might indicate that relative to the non-paretic side, the paretic limb segments may continue to lag in terms of angular position and velocity. Despite this, the increase in Sobolev norm and the modest correlation with 0.05–6 m/s improvements in gait speed indicate some gait improvement. A training protocol would be warranted, since LF individuals, in particular, may benefit more from a greater dosage or intensity in training [48].

Given the high correlation of Sobolev norm values (and intersegmental surface area) with gait speed, it remains to be seen if repeated sessions, as implemented during overground walking with the rehabilitation dog and cane [25], would be benefical to improveing these coordination outcomes along with gait speed. Despite these interesting findings, the Sobolev norm metric used in this study is still in an exploratory stage and requires further investigation before potentially implementing it as a clinical or diagnostic tool. In the process, it could also be further investigated in light of existing methods for reporting gait symmetry [49].

Several limitations should be considered in light of the encouraging findings. From a statistical perspective, the limited sample size and the stratification of LF and HF when dividing the target sample size of 14 into two equal groups should be noted when examining the inferential statistical results. This is especially pertinent considering the factor levels and the heterogeneity of the chronic stroke participants. In terms of possible methodological limitations, the cane condition was run separately from the leash trials and required average pre-force values of the leash conditions (10 and 15 N) for indirect change comparisons. Further, post-adaptation effects generally persisted during the entire post-force. It is therefore still unknown to what extent fatigue due to walking impacted the results. In terms of analysis the study addressed kinematic outcome variables and can be further substantiated by future studies that include a kinetic analysis underlying coordination change. A closer look at muscle activation as seen in other studies with light vibrotactile overground walking [43] would potentially offer more insight to the possible improvement of bilateral symmetry. Future studies may also want to factor the effects of the upper limb on future kinematic and dynamic analyses, since differences in arm swing may influence energy costs in gait [50], especially in the context of holding a leash, where bilateral arm positions and ROM differ. Lastly, in regard to functional gait and the recovery of symmetry, it would be interesting to investigate whether continued use of the haptic leash can further increase the ROM and angular velocity while ultimately reducing the discrepancy between non-paretic and paretic limb as potentially detected by 3D Phase diagrams and Sobolev norm values. Additionally, the high correlation seen in both intersegmental area and Sobolev norm values with respect to gait speed would suggest gains in functional gait and symmetry may potentially be achieved in tandem with further improvements in gait speed. The implementation of haptic leash training sessions involving a greater sample size could address this question.

## Conclusion

Changes in bilateral lower limb coordination accompanied gait speed increases when older chronic stroke and age-matched control individuals were exposed to a robot-powered haptic tensile force delivered in a VE. In general, the adaptation effects seen in coordination outcomes during the force epoch typically endured as post-adaptation effects when haptic forces were removed. Further kinematic analysis of lower limb coordination outlined slight increases in bilateral intersegmental surface area with some evidence of peak angle changes for an overall increase in ROM, which is potentially conducive to improved propulsion of the paretic limb. This was substantiated by some increases in angular velocity within the gait cycle, which were made visible in the 3D phase diagram. The Sobolev norm values assigned to each lower limb revealed relatively proportional increases of the paretic and non-paretic leg. Hence, while there was limited evidence for increased symmetry in a single walking session, potentially functional gait speed increases revealed high correlations with both intersegmental area and Sobolev norm outcomes. Consequently, the HF and CG groups showed more robust responses compared to the LF group. A haptic leash training protocol could be devised targeting the carryover effects observed, as such effects may well be conducive to retaining functional mobility and improving coordination in gait.

## Data Availability

All data reported in this manuscript was collected at the Feil and Oberfeld CRIR Research Center of the Jewish Rehabilitation Hospital in Laval, Québec, Canada. The data and supporting materials from this study can be made available by JF.

## Abbreviations

VR: Virtual reality
VE: Virtual environment
ROM: Range of motion
COM: Centre of mass
3D: 3-Dimensional
LF: Lower function post-stroke
HF: Higher function post-stroke
CG: Control group
PID: Proportional-integral-derivative
GEE: Generalized estimating equations
